# Predicting incident dementia in cerebral small vessel disease: comparison of machine learning and traditional statistical models

**DOI:** 10.1016/j.cccb.2023.100179

**Published:** 2023-08-09

**Authors:** Rui Li, Eric L. Harshfield, Steven Bell, Michael Burkhart, Anil M. Tuladhar, Saima Hilal, Daniel J. Tozer, Francesca M. Chappell, Stephen D.J. Makin, Jessica W. Lo, Joanna M. Wardlaw, Frank-Erik de Leeuw, Christopher Chen, Zoe Kourtzi, Hugh S. Markus

**Affiliations:** a Stroke Research Group, Department of Clinical Neurosciences, University of Cambridge, United Kingdom of Great Britain and Northern Ireland; b Heart and Lung Research Institute, University of Cambridge, United Kingdom of Great Britain and Northern Ireland; c Precision Breast Cancer Institute, Department of Oncology, University of Cambridge, United Kingdom of Great Britain and Northern Ireland; d Adaptive Brain Lab, Department of Psychology, University of Cambridge, United Kingdom of Great Britain and Northern Ireland; e Department of Neurology, Donders Centre for Medical Neuroscience, Radboud University Medical Centre, Nijmegen, Netherlands; f Memory Aging and Cognition Centre, Department of Pharmacology, Yong Loo Lin School of Medicine, National University of Singapore, Singapore; g Saw Swee Hock School of Public Health, National University of Singapore and National University Health System, Singapore; h Centre for Clinical Brain Sciences, University of Edinburgh, United Kingdom of Great Britain and Northern Ireland; i Centre for Rural Health, Institute of Applied Health Sciences, University of Aberdeen, United Kingdom of Great Britain and Northern Ireland; j Centre for Healthy Brain Ageing, University of New South Wales, Sydney, Australia

**Keywords:** Cerebral small vessel disease, Dementia, Prediction, Machine learning

## Abstract

**Background:**

Cerebral small vessel disease (SVD) contributes to 45% of dementia cases worldwide, yet we lack a reliable model for predicting dementia in SVD. Past attempts largely relied on traditional statistical approaches. Here, we investigated whether machine learning (ML) methods improved prediction of incident dementia in SVD from baseline SVD-related features over traditional statistical methods.

**Methods:**

We included three cohorts with varying SVD severity (RUN DMC, *n* = 503; SCANS, *n* = 121; HARMONISATION, *n* = 265). Baseline demographics, vascular risk factors, cognitive scores, and magnetic resonance imaging (MRI) features of SVD were used for prediction. We conducted both survival analysis and classification analysis predicting 3-year dementia risk. For each analysis, several ML methods were evaluated against standard Cox or logistic regression. Finally, we compared the feature importance ranked by different models.

**Results:**

We included 789 participants without missing data in the survival analysis, amongst whom 108 (13.7%) developed dementia during a median follow-up of 5.4 years. Excluding those censored before three years, we included 750 participants in the classification analysis, amongst whom 48 (6.4%) developed dementia by year 3. Comparing statistical and ML models, only regularised Cox/logistic regression outperformed their statistical counterparts overall, but not significantly so in survival analysis. Baseline cognition was highly predictive, and global cognition was the most important feature.

**Conclusions:**

When using baseline SVD-related features to predict dementia in SVD, the ML survival or classification models we evaluated brought little improvement over traditional statistical approaches. The benefits of ML should be evaluated with caution, especially given limited sample size and features.

## Highlights

• Evaluated ML improved little over statistical models in dementia prediction in SVD.
• Baseline global cognition is highly predictive of incident dementia in SVD.
• ML should be used with caution especially given limited sample size and features.

## Introduction

Cerebral small vessel disease (SVD) causes a quarter of ischaemic strokes and is the most common pathology underlying vascular dementia [1]. Furthermore, the presence of SVD increases the likelihood that other dementia pathologies, such as Alzheimer's disease, will cause clinically overt dementia [2]. Thus, SVD contributes to 45% of dementia cases worldwide [2] and presents an enormous global health challenge [3]. Typical manifestations of SVD on magnetic resonance imaging (MRI) include white matter hyperintensities (WMH), lacunes, enlarged perivascular space, and cerebral microbleeds (CMB) [4].

Despite the importance of SVD in the aetiology of dementia, only a minority of subjects with SVD develop dementia. For example, in a cohort with symptomatic lacunar stroke and confluent WMH, one fifth developed dementia during 5 years of follow-up [5]. Several factors are known to increase the risk of dementia, including age [6], low education [6], and SVD burden assessed on MRI––particularly lacune count, WMH volume, brain atrophy and the extent of white matter ultrastructural damage on diffusion tensor imaging (DTI) [5,[7], [8], [9]]. A number of approaches have been used to attempt dementia prediction in SVD. They include the use of a simple SVD severity score [10,11], and more advanced analysis of MRI [12]. In particular, DTI has been shown to be predictive of future dementia risk, and multiple ways of analysing the DTI metrics, such as the automatic marker, peak width of skeletonised mean diffusivity (PSMD), have been implemented [9,13].

Such models that can be applied on an individual patient basis to predict dementia risk in SVD are highly useful, both in providing prognostic information in the clinic, and in identifying patients to include in future clinical trials of potential treatments. However, most models targeting the SVD population to date have relied on traditional statistical approaches [11,14,15], whereas machine learning (ML) methods, which have fewer restrictive assumptions and can model more complex relationships among the predictors and outcome, are increasingly used for disease prediction in other settings including for Alzheimer's dementia (AD) [16]. Therefore, we sought to investigate whether common ML methods can improve prediction of incident dementia in SVD over traditional statistical approaches, when using baseline demographics, vascular risk factors, cognitive and MRI features related to SVD. To improve generalisability, we used data from three prospective cohort studies covering a wide range of SVD severity and ethnic diversity. We also addressed dementia prediction through both survival analyses utilising all follow-up time available, and classification analyses that predicted 3-year dementia risk. For both types of analyses, we trained and internally validated several ML and statistical models, using all or subsets of the selected feature modalities. Finally, we compared how different models ranked the importance of different input features in predicting dementia.

## Material and methods

We followed the Transparent Reporting of a Multivariable Prediction Model for Individual Prognosis or Diagnosis (TRIPOD) reporting guideline [17] (Table S1).

### Cohorts

Three prospective longitudinal cohort studies (Table 1) with multimodal MRI data, detailed neuropsychological assessment, and long-term follow-ups with dementia diagnoses recorded were included, which covered varying SVD severity. All participants were non-demented at baseline timepoint. Clinical diagnosis of mild cognitive impairment (MCI) at baseline was available in the HARMONISATION study, but not in RUN DMC or SCANS.

i **RUN DMC––**Radboud University Nijmegen Diffusion tensor and Magnetic resonance imaging Cohort study was a 9-year study with 503 predominantly mild symptomatic SVD patients, defined as presenting lacunes or WMH on neuroimaging and accompanying acute or subacute symptoms [19,20].
ii **SCANS––**St George's Cognition and Neuroimaging in Stroke study was a 5-year study with 121 severe symptomatic SVD patients, defined as having clinical lacunar stroke syndrome with confluent WMH on MRI [5,21].
iii **HARMONISATION––**A memory clinic study in Singapore that followed up 265 participants with clinically diagnosed MCI or normal cognition at baseline (MCI *n* = 127, normal cognition *n* = 115, unknown *n* = 23) for 5 years [22]. Although it was not originally an SVD cohort, it was included due to widespread imaging findings of SVD.

**Table 1:** Cohort information overview.

Cohort	RUN DMC	SCANS	HARMONISATION
**Baseline Cohort Size**	503	121	265
**Inclusion Criteria**	Symptomatic SVD, defined as the presence of lacunes or WMH on neuroimaging, and accompanying acute (lacunar stroke) or subacute (cognitive, motor) symptoms. Aged 50–85.	Symptomatic SVD, defined as a clinical lacunar stroke syndrome with MRI evidence of an anatomically corresponding lacunar infarct and confluent regions of WMH graded ≥ 2 on the modified Fazekas scale [18].	Combination of: • Patients with cognitive impairment but no dementia, defined as being impaired in at least one cognitive domain on a neuropsychological test battery without loss of daily functions. • Controls with no cognitive impairment on neuropsychological tests, or function loss, but may had subjective complaints of memory impairment.
**Centres**	Department of Neurology, Radboud University Nijmegen, The Netherlands	Stroke service at 3 hospitals in South London, UK	Memory clinics at National University Hospital and Saint Luke's Hospital, and adjacent community in Singapore
**Recruitment Period**	2006	12.2007–08.2010	08.2010–12.2016
**Follow-up Period**	Until 2015; 9 years.	Until 08.2015; 5 years.	Until 09.2020; 5 years.
**Assessments**	MRI and neuropsychological tests at baseline, year 5 and 9.	MRI at baseline, year 1,2,3. Neuropsychological tests at baseline, year 1,2,3,4,5.	MRI and neuropsychological tests at baseline, year 2,5.
**Dementia Diagnosis**	DSM-IV	DSM-V	DSM-IV

SVD = Cerebral small vessel disease; WMH= White matter hyperintensity; MRI = Magnetic resonance imaging; DSM = Diagnostic and Statistical Manual of Mental Disorders.

### Input features

Fifteen SVD-related features, which associate with incident dementia and were available in all cohorts, were selected as input to dementia prediction models. The complete list and descriptions are in Table S2. Briefly, by modality they were:

i Demographic features (*n* = 7)––Age, sex, years of education, and presence of vascular risk factors (hypertension, smoking, hypercholesterolaemia, diabetes mellitus), which increases dementia risk [6].
ii Imaging features (*n* = 5)––Total brain volume and typical SVD features on MRI that have been used for dementia prediction, including white matter lesion load (=WMH volume/total brain volume × 100%), lacune count, presence of CMB, and PSMD measured from DTI [9,11]. We used the presence, instead of count, of CMB due to the heavily skewed nature of the distribution and the ease of assessment. Nonetheless, we also analysed CMB count in sensitivity analyses, but it led to similar prediction performance.
iii Cognitive features (*n* = 3)––A global cognitive score and domain scores for executive function and processing speed, which are cognitive domains prominently impacted by SVD [12].

The demographic and imaging features were assessed as published previously [5,9,19,20,22]. Cognitive features were calculated from each cohort's neuropsychological assessment batteries (Table S3) as described in Supplementary Methods. We only used features measured at participants’ baseline visit. All continuous features were standardised before being input into prediction models.

### Outcome measure

The prediction outcome was incident all-cause dementia, diagnosed using Diagnostic and Statistical Manual of Mental Disorders [23] manual. We chose all-cause dementia instead of any dementia subtype, as this is a more clinically relevant outcome and accounts for the fact that most dementia patients have multiple underlying pathologies [24].

### Missing data

Three subjects developed dementia during the follow-up period, but their actual date of dementia diagnosis was missing. For these patients, the date of dementia diagnosis was estimated based on the midpoint between the study visits just before and after their dementia diagnosis, or the midpoint of the year of diagnosis if only the month and day were missing. After this minor imputation, 89% of all participants had all required variables including dementia outcome (Table S4). Assuming independence between missingness and outcome given the predictors, in which case complete-case analysis has negligible bias [25], we conducted complete-case analyses, thus excluding any cases with missing predictors or outcome. Additionally, we tested for significant differences in feature distribution between the original and selected samples, using two-sided *t-*test for continuous variables and χ^2^ test for categorical variables with *p*<0.05=significant.

### Overall analysis plan

We conducted both survival and classification analyses, where several ML algorithms were evaluated against Cox or logistic regression on common metrics. Using data pooled from three cohorts, we trained and tested all models under the same cross-validation setting. No gender-specific analyses were performed. Code was implemented in Python 3.10.0.

#### Survival analysis

For the survival analyses, we defined time-to-dementia as the time from baseline visit to recorded dementia diagnosis. Patients without a dementia diagnosis by last contact were censored. No competing risks were considered. We evaluated the following ML survival models, which represent the major types developed to date that are suitable for medium-sized samples and feature sets [26], against standard Cox proportional hazards model (CoxPH) [27]:

1 **Regularised CoxPH with Elastic Net Penalty (Reg_Cox)**

This is CoxPH with regularised model coefficients to improve model's generalisability. We classified this model as ML following the practice in [26,28], though this might be debatable, as regularisation does not change the model structure.

2 **Random Survival Forests (RSF)**[29]

This is a nonlinear ensemble model consisting of independent survival trees built in parallel.

3 **Gradient Boosted Survival Trees (GBT)**[30]

This is another nonlinear ensemble model with shallow survival trees built sequentially.

Details about models and implementation are in Supplemental Methods and Table S5. The models were evaluated on concordance index (C-index) [31] measuring model discrimination, and the integrated Brier score (IBS) [32] measuring the overall error in predicted survival (so smaller values=better). We conducted Schoenfeld residuals tests in the pooled dataset and found no violation of the proportional-hazard assumption.

#### Classification analysis

For the classification analyses, we predicted whether individuals would develop dementia within three years of baseline, thus classifying the patients into two categories. An individual's 3-year dementia status was determined as described in the Supplemental Methods. The 3-year window was chosen to maximise the sample size (as those censored before year 3 were excluded due to unknown outcome) while maintaining clinical relevance. We evaluated the following ML classification algorithms against standard logistic regression:

1 **Regularised Logistic Regression with Elastic Net Penalty (Reg_Logistic)**

This is logistic regression with regularised model coefficients for better generalisability to unseen data. As with Reg_Cox, we classified this model as ML, though this might be debatable.

2 **Support Vector Machine (SVM)**[33]

As one of the most popular ML methods in dementia prediction [34], an SVM classifier finds a class boundary with maximum distance to the nearest datapoints from each class. We experimented with both linear and radial basis function kernels for SVM, which enabled it to learn linear and nonlinear class boundaries respectively.

3 **Generalised Matrix Learning Vector Quantisation (GMLVQ)**[35]

Chosen due to promising performance in predicting Alzheimer's dementia [16], a GMLVQ model learns class prototypes and a full relevance matrix, which encodes the relative importance of and interactions between input features for prediction.

4 **Generalised Relevance Learning Vector Quantisation (GRLVQ)**[35]

Unlike GMLVQ, GRLVQ learns a diagonal relevance matrix––it ignores interactions between features but is less data-hungry to train.

Details about models and implementation are in Supplemental Methods and Table S5. The models were evaluated using area under the receiver operating curve (ROC-AUC), accuracy, sensitivity, specificity, precision, and G-mean (=$\sqrt{sensitivity\times specificity}$), which measures balanced performance on two classes.

#### Model training and evaluation

We pooled data from three cohorts and trained and tested each prediction model in a nested 5-fold cross-validation framework (Supplemental Methods), which incorporates hyperparameter tuning in an automatic and unbiased way [36]. Where hyperparameter tuning was not needed, simple 5-fold cross validation was used instead. All fold-splitting was stratified by both dementia outcome and cohort, so that the proportion of dementia cases and the ratio of subjects from different cohorts were identical for training/validation/test sets. We used paired *t-*test to compare the test results of each ML model and its statistical counterpart. After finishing the cross validation, a final model was trained for each algorithm on the entire pooled dataset.

To counter the class imbalance problem in the classification analysis (only 6.4% of all participants developed dementia within 3 years), we randomly oversampled cases with dementia during training to increase their weights and to achieve comparable performance between two classes during testing. Other interpolation-based oversampling techniques [37] were also tried but did not improve further. All oversamplers were implemented with Python imbalanced-learn package [38].

#### Feature importance analysis

Apart from using all selected features, we also experimented with using subsets of feature modalities. Since demographic features are easily attainable in clinical practice, we ran models using only demographic features, or using demographics in combination with imaging and/or cognitive features.

Furthermore, we examined how each prediction algorithm ranked the relative importance of the 15 selected features. For the standard/regularised regression models and the LVQ models, this could be interpreted straightforwardly from their final models trained with all features on the entire dataset––we examined the magnitude of the *β*-coefficients of the regression models and the diagonal elements of the relevance matrices of the LVQ models.

Other models (including SVM due to the selection of the radial basis function kernel from nested cross validation) did not allow direct interpretation of feature importance as above. Instead, we removed each feature from the complete feature set one at a time and evaluated the reduction in average test ROC-AUC or C-index from cross-validation experiments. Features causing greater reductions were considered to be more important.

#### Data and code availability

Data from the three cohorts is available to *bona fide* researchers upon reasonable request, subject to approval of the relevant regulatory and ethical bodies. Code for all analyses reported is available at https://gitlab.developers.cam.ac.uk/stroke/papers/rui-dementia-prediction-in-svd-with-ml.

## Results

### Cohort data characteristics

We had 889 participants from the three cohorts at baseline. After excluding those with missing data (Table S4), 789 participants (46.3% females, mean age [SD] = 67.62 [9.10]) were included in our survival analysis, of which 108/789 (13.7%) were diagnosed with dementia during a median (IQR) follow-up period of 5.4 (4.1, 8.7) years (Table 2). The RUN DMC cohort was younger (mean age=65) than SCANS (mean age=70) and HARMONISATION (mean age=71) on average. It also had the lowest proportion of dementia cases (11.4%; SCANS=17.3%; HARMONISATION=16.2%) despite having the longest follow-up. In the classification analysis, after further excluding those censored before three years due to unknown outcome, 750 participants were included, amongst whom 48/750 (6.4%) developed dementia within 3 years. No significant difference in the distribution of input features was found between the original pooled data and the data included in either analysis.

**Table 2:** Cohort characteristics.

Cohort	RUN DMC	SCANS	HARMONISATION	POOLED
Sample size in survival analysis	439	110	240	789
No. dementia cases in survival analysis	50 (11.4%)	19 (17.3%)	39 (16.2%)	108 (13.7%)
Cases per dementia subtype in survival analysis	28 AD 14 VD 6 mixed AD/VD 1 LBD 1 Unknown	19 VD	34 AD 5 VD	62 AD 38 VD 6 mixed AD/VD 1 LBD 1 Unknown
Follow-up years *Median (IQR)*	8.7 (8.5, 8.9)	5.0 (4.0, 5.1)	4.0 (3.0, 5.0)	5.4 (4.1, 8.7)
Sample size in classification analysis	429	98	223	750
No. dementia cases (by year 3) in classification analysis	14 (3.3%)	10 (10.2%)	24 (10.8%)	48 (6.4%)
Cases per dementia subtype in classification analysis	11 AD 2 VD 1 Unknown	10 VD	22 AD 2 VD	33 AD 14 VD 1 Unknown
Input Features*				
Age *Mean (SD)*	65.17 (8.87)	70.01 (9.93)	70.98 (7.61)	67.62 (9.10)
Years of Education *Mean (SD)*	10.88 (3.55)	11.56 (3.49)	8.45 (4.90)	10.24 (4.18)
Sex *(Female%)*	196 (44.6%)	39 (35.5%)	130 (54.2%)	365 (46.3%)
Hypertension *(Yes%)*	317 (72.2%)	101 (91.8%)	159 (66.2%)	577 (73.1%)
Hypercholesterolaemia *(Yes%)*	194 (44.2%)	95 (86.4%)	185 (77.1%)	474 (60.1%)
Diabetes mellitus *(Yes%)*	52 (11.8%)	21 (19.1%)	71 (29.6%)	144 (18.3%)
Smoking *(Yes%)*	307 (69.9%)	67 (60.9%)	53 (22.1%)	427 (54.1%)
White matter lesion load *Median (IQR)*	0.29 (0.10, 0.98)	3.06 (1.60, 4.54)	0.24 (0.05, 0.81)	0.37 (0.11, 1.42)
Lacune count *Median (IQR)*	0 (0, 0)	2 (0, 5)	0 (0, 1)	0 (0, 1)
Presence of cerebral microbleeds *(Yes%)*	69 (15.7%)	43 (39.1%)	89 (37.1%)	201 (25.5%)
Total brain volume (ml) *Mean (SD)*	1069.13 (77.22)	1043.10 (104.14)	896.86 (96.40)	1013.10 (116.78)
PSMD *Mean (SD)*	3.46 × 10^−4^ (7.55 × 10^−5^)	3.77 × 10^−4^ (1.11 × 10^−4^)	3.40 × 10^−4^ (7.31 × 10^−5^)	3.49 × 10^−4^ (8.16 × 10^−5^)
Global cognition *Mean (SD)*	0.03 (0.73)	−0.65 (0.81)	−0.56 (0.82)	−0.25 (0.83)
Executive function *Mean (SD)*	0.02 (0.76)	−0.86 (1.05)	−0.68 (1.45)	−0.32 (1.12)
Processing speed *Mean (SD)*	0.04 (0.85)	−0.99 (0.88)	−0.51 (1.05)	−0.27 (1.00)

AD = Alzheimer's dementia; VD = Vascular dementia; LBD = Lewy body dementia; IQR = Interquartile range (25%, 75%); SD = Standard deviation; PSMD = Peak width of skeletonised mean diffusivity.

⁎ Statistics of the input features are for the sample included in the survival analysis.

### Test performance of survival models

**Fig. 1:**
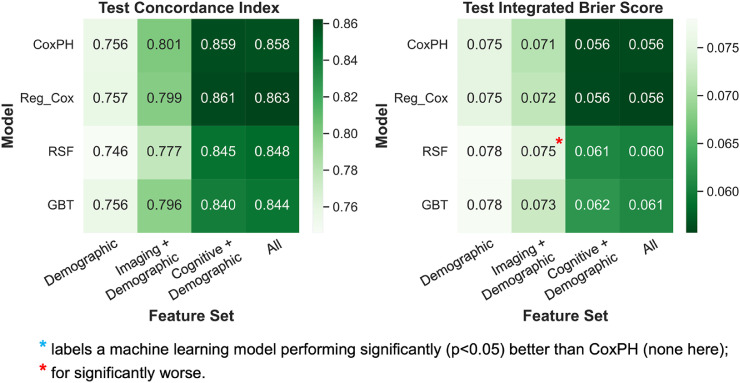
Heatmaps showing the average test performance of each survival model-feature set combination from cross-validation experiments. **Survival models**: CoxPH = Cox proportional hazards model; Reg_Cox = Regularised CoxPH model with elastic net penalty; RSF = Random survival forests; GBT = Gradient boosted survival trees.

All survival models performed well and achieved average test C-indices above 0.8 when using all features – CoxPH, 0.858; Reg_Cox, 0.863; RSF, 0.848; GBT, 0.844 (Fig. 1, S1, Table S6). However, there was little improvement in performance by any ML method when compared with traditional CoxPH.

Overall, the regularised Cox model achieved slightly higher C-indices than standard CoxPH with most feature sets, but the differences remained insignificant, and Reg_Cox showed no improvement in IBS. Both RSF and GBT models underperformed CoxPH on average with all feature sets, though they have more stable performance (Figure S1). Results from both training and testing (Figure S1) showed clear signs of overfitting for the RSF model only, as indicated by much better training performance than testing.

### Test performance of classification models

**Fig. 2:**
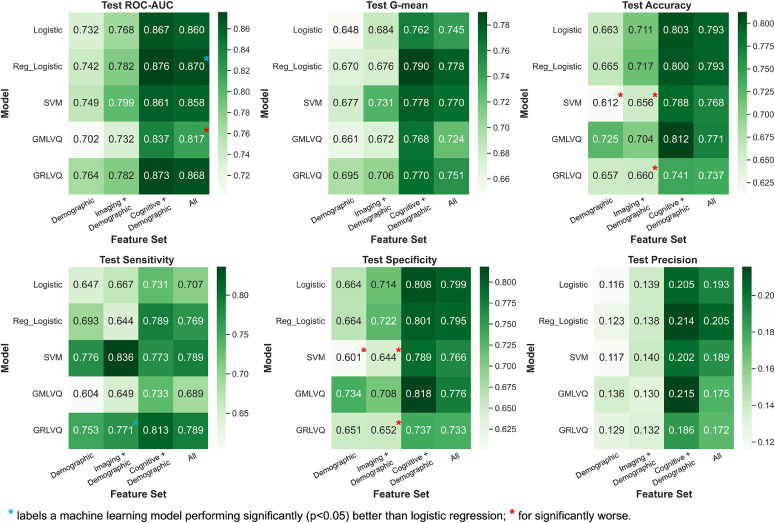
Heatmaps showing the average test performance of each classification model-feature set combination from cross-validation experiments. **Classification models**: Logistic = Logistic regression; Reg_Logistic = Regularised logistic regression with elastic net penalty; SVM = Support vector machine; GMLVQ = Generalised matrix learning vector quantisation; GRLVQ = Generalised relevance learning vector quantisation.

Similar to that observed in the survival analysis, all classification models performed reasonably well, scoring average test ROC-AUC above 0.8 when using all features – Logistic, 0.860; Reg_Logistic, 0.870; SVM, 0.858; GMLVQ, 0.817; GRLVQ, 0.868 (Fig. 2, S2, Table S7). However, most ML models did not significantly outperform standard logistic regression for any feature set.

The only model that consistently scored better than or similarly to logistic regression on all metrics was regularised logistic regression. It achieved an ROC-AUC of 0.870±0.061 when using all features, which was significantly (*p* = 0.019) higher than logistic regression (0.860±0.065). GRLVQ appeared to be the second-best model overall, with higher (though non-significantly) ROC-AUC and G-mean than logistic regression on average. However, GRLVQ had significantly worse specificity (0.652±0.042) and accuracy (0.660±0.038) than logistic regression (specificity, 0.714±0.042; accuracy, 0.711±0.038) when only imaging and demographic features were used. This pattern of better sensitivity but worse specificity than logistic regression was also observed for the SVM model, but the opposite was true for GMLVQ. Both SVM and GMLVQ underperformed logistic regression overall, with only comparable or significantly worse metrics than logistic regression for any feature set.

### Results from feature importance analysis

In the cross-validation results from both survival and classification analyses (Figs. 1,2), we observed for all models that using only demographic and cognitive features led to similar, or sometimes even better, results than using all features from three modalities. In other words, adding the imaging variables did not improve prediction further. However, when excluding the cognitive data, adding imaging features to demographics alone still improved the prediction of most models.

**Fig. 3:**
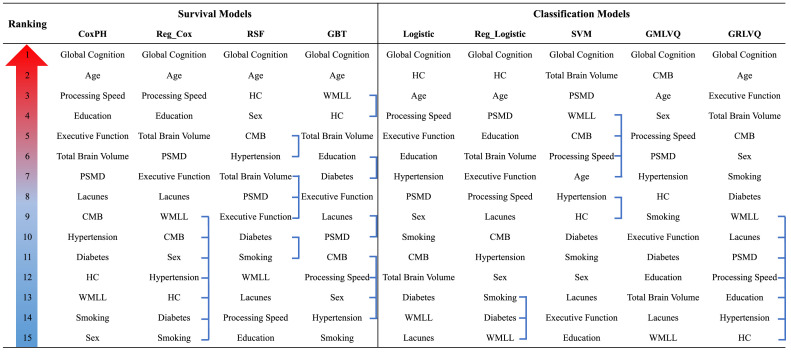
Rankings of feature importance by each survival and classification model. Linked features have tied rankings. **Models:** CoxPH = Cox proportional hazards model; Reg_Cox = Regularised CoxPH model with elastic net penalty; RSF = Random survival forests; GBT = Gradient boosted survival trees; Logistic = Logistic regression; Reg_Logistic = Regularised logistic regression with elastic net penalty; SVM = Support vector machine; GMLVQ = Generalised matrix learning vector quantisation; GRLVQ = Generalised relevance learning vector quantisation. **Input Features**: PSMD = Peak width of skeletonised mean diffusivity; CMB = Presence of cerebral microbleeds; HC = hypercholesterolaemia; WMLL = White matter lesion load.

The rankings of all input features by each prediction model are shown in Fig. 3 (also Tables S8,S9). Despite the differences in model type, all models consistently ranked global cognition as the most important feature, which agreed with the importance of cognitive features observed previously. All survival models were also consistent in choosing the second most important feature, age, which also always remained amongst the top five features selected by the classification models. Apart from these, there was less agreement among the models on the rankings for other features. However, the two best performing survival and classification models, regularised CoxPH and regularised logistic regression, both selected PSMD and TBV as the two most important imaging features. Besides, we observed in Table S8, S9 that the few least important features for the regularised Cox and logistic regression and GRLVQ models had zero coefficients/weights – these features were effectively disregarded by the models.

## Discussion

Advances in machine learning have increasing led to its use in dementia prediction. In this study of 789 participants from three cohorts with SVD, we investigated whether common ML methods can better predict incident dementia in SVD than traditional statistical methods when using baseline SVD-related features.

Both our survival and classification analyses showed very limited improvement by ML methods over statistical approaches, though most models performed well. In both analyses, only regularised Cox/logistic regression outperformed their statistical counterparts in testing, though the improvement was insignificant in the survival context. Other more complex ML survival or classification models achieved even worse or comparable performance than the statistical models on average. We also determined the relative contribution of different features to prediction and found adding imaging features when cognitive features were included brought little improvement. Global cognition was the most predictive feature to all models.

Our results showing limited improvement by ML are consistent with conclusions from a systematic review [39] and other comparative studies, especially when similar numbers of features are selected [26,40,41]. The observed benefit from regularisation also agrees with past literature [26], and the benefit might come from the fact that the regularisation minimised the influence of noisy, less important features by shrinking their coefficients down to zero. These regularised models also had much fewer parameters to train compared with other ML models, which was desirable given the limited sample size.

On the other hand, the worse performance of other more complex, nonlinear ML models in comparison to linear Cox/logistic regression may suggest a genuine linear relationship amongst the features for predicting dementia, which was better captured by the linear models. This might be true given the relatively small number of features included. In addition, our datasets might not have been large enough to train some of these ML models, which would have led to overfitting and suboptimal performance in testing – this was the case for the RSF model, but other ML models did show reasonable generalisability from training to internal testing.

Moreover, our feature importance analyses showed the predominant importance of current cognition in dementia prediction. However, the cognitive assessments in these cohorts were time consuming, taking 1–2 h. Therefore, prediction based on demographic and imaging data alone could provide an alternative approach in clinical practice. Whether simple cognitive assessments using a short screening battery are equally predictive remains an important unresolved clinical question.

Our study has several strengths. First, we used a relatively large dataset from multiple cohorts encompassing a broad range of SVD severity and ethnic diversity. Second, we conducted both survival and classification analyses that arrived at similar conclusions, thereby reducing the potential for bias that could be imposed by using only one analysis type. Moreover, we evaluated a variety of ML models with different underlying structure, which strengthened the robustness of the findings.

However, there were also limitations. First, our focus in this analysis was on well-established SVD features, which were relatively limited. For the prediction of all-cause dementia, the inclusion of other markers such as regional cortical brain volumes, particularly hippocampal volumes, might improve prediction for all models, because those markers can reflect the pathological processes of cortical dementias, which may act additively with SVD changes to increase the chance of mixed dementia. Furthermore, it is possible that with higher dimensional data (i.e., more input variables, such as using the original MRI images on a voxel-by-voxel basis), in which the relationships amongst variables are more complex, advanced ML methods may better model these relationships and confer greater advantages in predicting dementia in SVD populations, though this remains to be evaluated in future studies.

Other limitations include differences in the MRI and cognitive assessment protocols across cohorts, but despite these inconsistencies, cognitive features still exhibited high predictive value. Furthermore, our approach of addressing class imbalance in the classification analysis may not have been ideal [39], though we applied the same oversampling to all classification models to maintain fairness. Finally, we did not perform external validation, so it is not known to what extent our findings are generalizable to other cohorts.

## Conclusions

When using baseline SVD-related features to predict incident dementia in SVD, the ML survival or classification models we evaluated brought little improvement over traditional statistical approaches. Our results suggest the use of Cox or logistic regression with regularisation. In general, the prediction model should be chosen carefully for each project based on the sample size, predictors available and requirements on interpretability. The benefits of ML should be evaluated with caution especially when using small-to-medium-sized datasets with a limited number of features.

## Acknowledgements

The authors disclosed receipt of the following financial support for the research, authorship, and/or publication of this article: This research was funded by a British Heart Foundation (BHF) programme grant [grant number RG/F/22/110052] and infrastructural support was provided by the Cambridge British Heart Foundation Centre of Research Excellence [grant number RE/18/1/34212] and the Cambridge University Hospitals NIHR Biomedical Research Centre [grant number BRC-1215–20014].

HSM is supported by an NIHR Senior Investigator Award, and a number of peer reviewed funders including Medical Research Council, EU, Alzheimer's Society, Stroke Association, BHF. The views expressed are those of the authors and not necessarily those of the NIHR or the Department of Health and Social Care. RL is supported by a PhD scholarship awarded by Trinity College, University of Cambridge. ELH is supported by Cambridge BHF Centre of Research Excellence [grant number RE/18/1/34212]; Alzheimer's Society [grant number AS-RF-21–017]; BHF programme grant [grant number RG/F/22/110052]; Cambridge NIHR Biomedical Research Centre [grant number BRC-1215–20014]. SB is supported by 10.13039/501100000274BHF. AMT is supported by 10.13039/501100002996Dutch Heart Foundation [grant number 2016T044]. Wellcome Trust [grant number 081589] provided initial funding for SCANS study. SDJM is supported by 10.13039/100010269Wellcome Trust [grant number WT088134/Z/09/A]. JMW is supported by 10.13039/100010269Wellcome Trust, Row Fogo Trust, and Medical Research Council. CC is supported by 10.13039/501100001349National Medical Research Council of Singapore. ZK is supported by 10.13039/100010269Wellcome Trust and Alan Turing Institute.

## Ethical approval & informed consent

Each cohort study obtained local ethical approval and written informed consent from participants.

## CRediT authorship contribution statement

**Rui Li:** Methodology, Data curation, Writing – review & editing. **Eric L. Harshfield:** Conceptualization, Methodology. **Steven Bell:** Data curation. **Michael Burkhart:** Data curation. **Anil M. Tuladhar:** Data curation. **Saima Hilal:** Data curation. **Daniel J. Tozer:** Data curation. **Francesca M. Chappell:** Data curation. **Stephen D.J. Makin:** Data curation. **Jessica W. Lo:** Data curation. **Joanna M. Wardlaw:** Data curation. **Frank-Erik de Leeuw:** Data curation. **Christopher Chen:** Data curation. **Zoe Kourtzi:** Data curation. **Hugh S. Markus:** Conceptualization, Methodology, Supervision, Writing – review & editing.

## Declaration of Competing Interest

The authors declare that they have no known competing financial interests or personal relationships that could have appeared to influence the work reported in this paper.

## Appendix Supplementary materials

Image, application 1
