# Supplementary material for: Predicting incident dementia in cerebral small vessel disease: comparison of machine learning and traditional statistical models
Source: Cereb Circ Cogn Behav. 2023 Aug 9;5:100179. doi: 10.1016/j.cccb.2023.100179 (PMC10428032; doi:10.1016/j.cccb.2023.100179)
Supplement: Supplementary file 1 [file mmc1.docx]

**Supplementary Materials**

**Supplemental Methods**

**Calculation of Cognitive Features**

In each cohort, the raw score from each test was firstly standardised (z-scored) with regard to age-matched healthy controls where possible, using the formula $z={(value-mean)}/{SD}$ —thus, the mean and standard deviation (SD) are those of healthy controls on the corresponding tests, and the z-scores indicate how much each participant deviate from the healthy ‘norm’ in each test. Specifically, in SCANS, the mean and SD for each test were taken from the best available published age-matched normative data;^1^ in HARMONISATION, the mean and SD were those of the control group in the study; but in RUN DMC, mean and SD of the whole sample at baseline timepoint were used, due to the lack of age-matched normative data. *Cognitive domain scores* (for executive function and processing speed) were then calculated as the average of the z-scores of the subset of tests measuring each domain (**Table S3**). *Global cognition* was calculated as the average of the z-scores of all tests used in each cohort.

**Labelling 3-Year Dementia Outcome**

Given that the recorded date of dementia diagnosis was only an approximate estimate of when the disease was developed, when labelling the 3-year dementia outcome for patients, we firstly rounded the time-to-dementia/censoring of each patient to its nearest integer, and then labelled those with rounded time-to-dementia ≤ 3 years as the positive (dementia) class, and those with rounded time-to-censoring ≥ 3 years as the negative (dementia-free) class. We also excluded those participants with rounded time-to-censoring less than 3 years, as whether they were diagnosed with dementia by 3 years was unknown. In this way, those patients who developed dementia at e.g. 3.1 years post baseline were still labelled as the positive class, and those who were censored just before the end of year 3 did not have to be excluded from analyses.

**Description of Survival Models**

***Cox Proportional Hazards Model (CoxPH)***

The Cox proportional hazards model^2^, also known as Cox regression, is the most widely used multivariable survival model. In its simplest form, which was what we used, the hazard function, *h(t),* of an individual with M features *x_1_*, *x_2_, … x_M_* is modelled by Equation (1). *h_0_(t)* is a baseline hazard function that changes only with time, and it can be estimated by Breslow estimator. *h_0_(t)* is the same for all individuals, so their hazard functions are proportional to each other and to the baseline hazard, hence the name of the model. The model coefficients, *β*s, are found by maximising the Cox partial likelihood function during training.

$h\left( t \right)=h_{0}(t)e^{\beta_{1}x_{1}+\beta_{2}x_{2}+\ldots+\beta_{M}\beta_{M}}$ (1)

The CoxPH model is semi-parametric, as the exponential part is fully specified, but the baseline hazard function is not parameterised. The standard form shown above is also a linear model that ignores inter-feature interactions, but we can also include nonlinear terms of different features and feature with time in the exponential, though the exact form needs to be explicitly specified. Additionally, the proportional hazard assumption may not always be satisfied, but this assumption can be tested using, for example, a Schoenfeld residuals test.

***Regularised CoxPH with Elastic Net Penalty***

This reduces the magnitude of the coefficients (*β*s) of a CoxPH model by adding elastic net penalty to its loss function, Cox partial likelihood function, during training, so that *β*s are found by minimising the new loss function defined by Equation (2). The elastic net penalty term is a combination of L_1_ and L_2_ penalty – L_1_ penalty is also known as the LASSO penalty, which is the sum of the absolute values of model coefficients and tends to reduce the coefficients of less important features down to zero, thus effectively eliminating those features; L_2_ penalty is also called the Ridge penalty, which is the sum of squared model coefficients and does not eliminate features. The hyperparameters, *α* and *r*, control the amount and ratio of the two types of penalty respectively. We tuned them through nested cross validation, to benefit from both types of regularisation. By regularising CoxPH, we prevent the model from making over-confident predictions and reduce the impact of less important features, which may improve the model’s generalisability to unseen data.

$arg\min_{\beta} Loss+\alpha(r\sum_{i=1}^{M} \left| \beta_{i} \right|+\frac{1-r}{2}\sum_{i=1}^{M} \beta_{i}^{2})$ (2)

***Random Survival Forests (RSF)***

The random survival forests^3^ (RSF) algorithm is an extension of the random forests machine learning algorithm to survival analysis. Briefly, an RSF model is an ensemble of survival trees, each trained on an independent bootstrap sample of the training dataset with the same sample size. At each node of the survival tree, a subset of the features will be randomly selected, and among these, the feature variable that maximizes the survival difference between the daughter branches will be used for splitting. Each tree can be grown only up to a pre-specified maximum depth, and it predicts a cumulative hazard function, $H\left( t \right)=\int_{0}^{t} h(t)$. The final model’s predicted H(t) is an average of those from all survival trees. The number of survival trees, number of candidate features to consider at each node, and the maximum tree depth were the hyperparameters that we optimised over.

In contrast to CoxPH, RSF is non-parametric and can handle much higher-dimensional data and more complex nonlinear relationships without requiring explicit specification of the form of the nonlinear terms. It also does not assume proportional hazards. The two levels of randomness at both bootstrapping the samples and selecting candidate features for each node are to improve the generalisability of the ensemble model. However, the RSF model does have many more parameters than CoxPH to train and therefore typically requires a larger training set.

***Gradient Boosted Survival Trees (GBT)***

This is an adaptation of the gradient boosting algorithm^4^ to survival analysis, and it also constructs an ensemble of short survival trees/stumps (depth=1), which are referred to as the base learners. However, unlike the random survival forest algorithm, the GBT constructs the survival stumps sequentially in a greedy stagewise fashion, with each stump added to correct the residuals in loss of the ensemble model in the previous iteration. The final model output is the added result of the base learners. The loss function we used here was Cox partial likelihood function. The number of base learners is the hyperparameter that we optimised over.

**Description of Classification Models**

***Logistic Regression***

Given *M* scalar features *x_1,_ x_2_, …, x_M_* of a subject, a standard logistic regression model computes the probability that this subject belongs to the positive class by Equation (3), where *y* is the binary class label and *β*s are model coefficients. This simplest form of logistic regression, which was what we used in our experiments, produces a linear class boundary, and does not consider nonlinear interactions between features. Note that, however, you can model nonlinear interactions by adding covariates that are nonlinear combinations of features, such as the product of two features, into the model, but this needs to be explicitly specified and manually tuned.

$P\left( y=1 \right)= \frac{1}{1+e^{-(\beta_{0}+\beta_{1}x_{1}+\beta_{2}x_{2}+\ldots+\beta_{M}\beta_{M})}}$ (3)

***Regularised Logistic Regression with Elastic Net Penalty***

Just as for the regularised CoxPH model described above, this regularised logistic regression model adds elastic net penalty on model coefficients to its original loss function during training, which should improve the model’s generalisability. We also optimised over the hyperparameters α and r, which control the amount and ratio of L_1_ and L_2_ penalty respectively.

***Support Vector Machine (SVM)***

Support vector machine^5^ classifiers are max-margin classifiers. In other words, they find the class boundary that has the maximum margin, or distance, to the nearest datapoints from each class, thus reducing the chance of overfitting. To cope with outliers, a certain amount of misclassification can also be allowed by adjusting a regularising hyperparameter, C. A higher value of C allows less misclassification, but also requires the class boundary to fit to the training data better, which may result in a smaller margin and poorer generalisability. Compared with logistic regression, SVMs can work better with higher-dimensional data, even when the number of dimensions (features) exceeds the number of samples.

The simplest form of SVM is a linear model, which learns a linear class boundary just like logistic regression. This is referred to as SVM with linear kernel, and its perceived feature importance can be directly interpreted by examining the coefficients associated with each feature. However, SVMs can also be made much more versatile through using various kernel functions. This essentially expands the feature dimension with nonlinear terms of different training datapoints, so that a nonlinear class boundary can be constructed. One of the most popular kernels is the radial basis function (RBF), which has a Gaussian form as shown in Equation (4). ***x*** and ***x'*** are different datapoints, and the hyperparameter *γ* controls the radius of the kernel, i.e. how far a datapoint can affect others. The feature importance in an SVM model with nonlinear kernels are not directly interpretable.

$RBF=e^{-\gamma\left\| \boldsymbol{x}-\boldsymbol{x'} \right\|^{2}}$ (4)

***Generalised Matrix Learning Vector Quantisation (GMLVQ)***

Generalised Matrix Learning Vector Quantisation (GMLVQ)^6^ belongs to the broader Learning Vector Quantisation classification scheme, which learns class prototypes in a supervised manner and makes predictions based on distances from new datapoints to prototypes. Briefly, it works in the following way for binary classification:

Suppose we have a training dataset in which each subject is represented by an M-dimensional input feature vector, ***x***, and a binary class label, *y.* A GMLVQ model finds the class prototypes, which can be understood as the typical datapoints of each class, by minimising the average generalised distance between the prototypes and their class instances. The generalised distance between an instance, ***x***, and a class prototype, ***w***, is defined by Equation (5), where **Λ** is a full M×M matrix called the relevance matrix. **Λ** is made symmetric and semi-positive definite through enforcing $\boldsymbol{\Lambda=}\boldsymbol{\Omega}^{T}\boldsymbol{\Omega}$.

$d_{\Lambda}\left( \boldsymbol{x, w} \right)=\left( \boldsymbol{x-w} \right)^{T}\boldsymbol{\Lambda}\left( \boldsymbol{x-w} \right)$ (5)

$\boldsymbol{=}\left( \boldsymbol{x-w} \right)^{T}\boldsymbol{\Omega}^{T}\boldsymbol{\Omega}\left( \boldsymbol{x-w} \right)\boldsymbol{\geq}0$

Training a GMLVQ model involves learning both the class prototypes and the relevance matrix, and the number of prototypes for each class is a hyperparameter that we optimised over. In prediction, a datapoint is assigned the same class as that of its closest prototype as measured by the generalised distance. Note that as the same **Λ** is used for each class in a GMLVQ model, the class boundary is piecewise linear, but it is overall nonlinear when more than a single class prototype is learnt for both classes.

The GMLVQ model is highly interpretable, as the diagonal terms of the relevance matrix **Λ** indicate the relative importance of each feature, and the off-diagonal terms indicate the pairwise interaction between different features in class prediction. The learnt class prototypes can be also examined to discover what the model has learned about each class.

***Generalised Relevance Learning Vector Quantisation (GRLVQ)***

The GRLVQ^6^ model is largely the same as the GMLVQ model, except that it uses a diagonal relevance matrix, **Λ**, instead of a full one. Eliminating the off-diagonal terms means that the model does not consider the interactions between different features when making prediction. This can limit its power, but it also gives it much fewer parameters to train, especially when there are more than a couple of features. This may be beneficial when the sample size is small.

**Nested Cross Validation**

Nested cross validation^7^ (CV) incorporates hyperparameter optimisation into the cross-validation framework, while avoiding data leakage and subsequent bias. As shown in the diagram below, a 5-fold nested CV starts with an outer-loop CV that splits the entire pooled dataset into 5 folds. Each fold takes turns to be reserved for testing, while the other folds are further used by an inner-loop 5-fold CV for hyperparameter optimisation – specifically, with each hyperparameter setting, we train a model on the training folds and test on the validation fold. After finishing the inner-loop CV, we select the hyperparameters that gave the best average validation results. With these, we train a new model on all data used in the inner loop, and test it on the reserved test fold.

After finishing the nested CV, each model/algorithm will have 5 test results. We can also train a final model on the entire pooled dataset by repeating what we did in the inner loop CV. This final model trained with all data available can then be tested externally if needed.


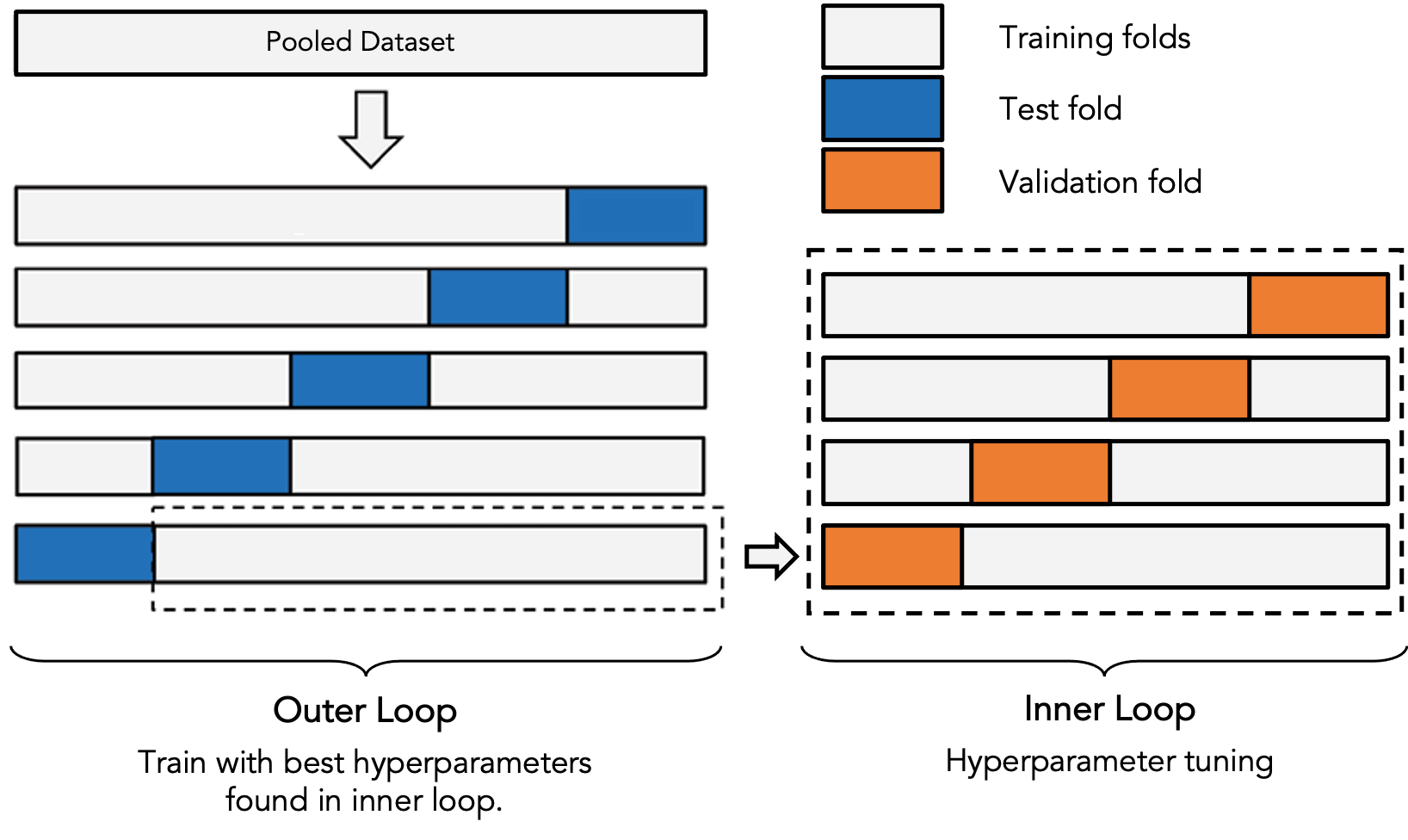


Supplemental Method Figure 1 — Diagram of Nested Cross Validation

**Supplemental Figures**

**Figure S1** – Box plots of the training and testing performance of all investigated survival models in 5-fold nested cross validation


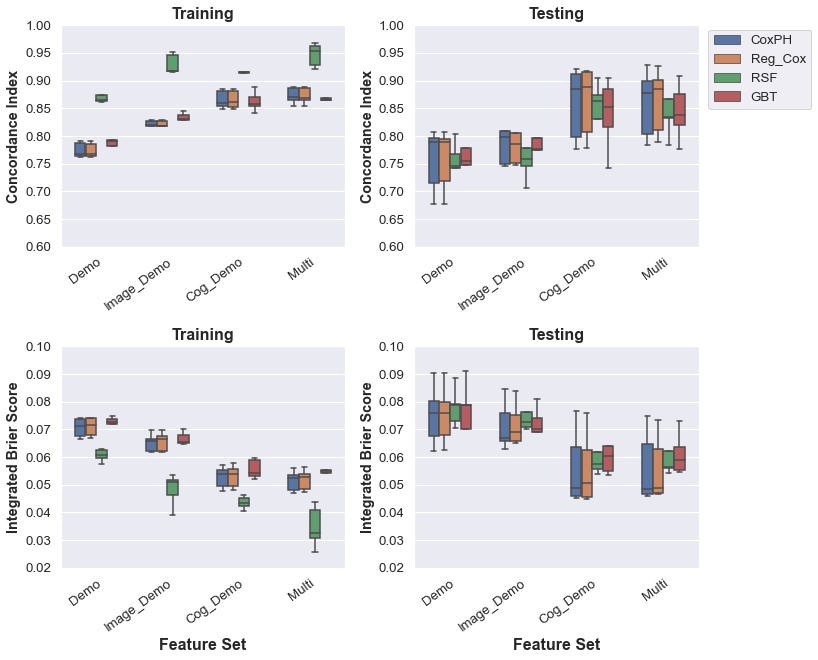


**Survival models**: CoxPH = Cox proportional hazards model; Reg_Cox = Regularised CoxPH model with elastic net penalty; RSF = Random survival forests; GBT = Gradient boosted survival trees.

**Feature sets**: Demo = Demographic feature set (N=7); Image_Demo = Imaging and demographic feature set (N=12); Cog_Demo = Cognitive and demographic feature set (N=10); Multi = Multimodal feature set (N=15).

**Figure S2** – Box plots of the training and testing performance of all investigated classification models in 5-fold nested cross validation


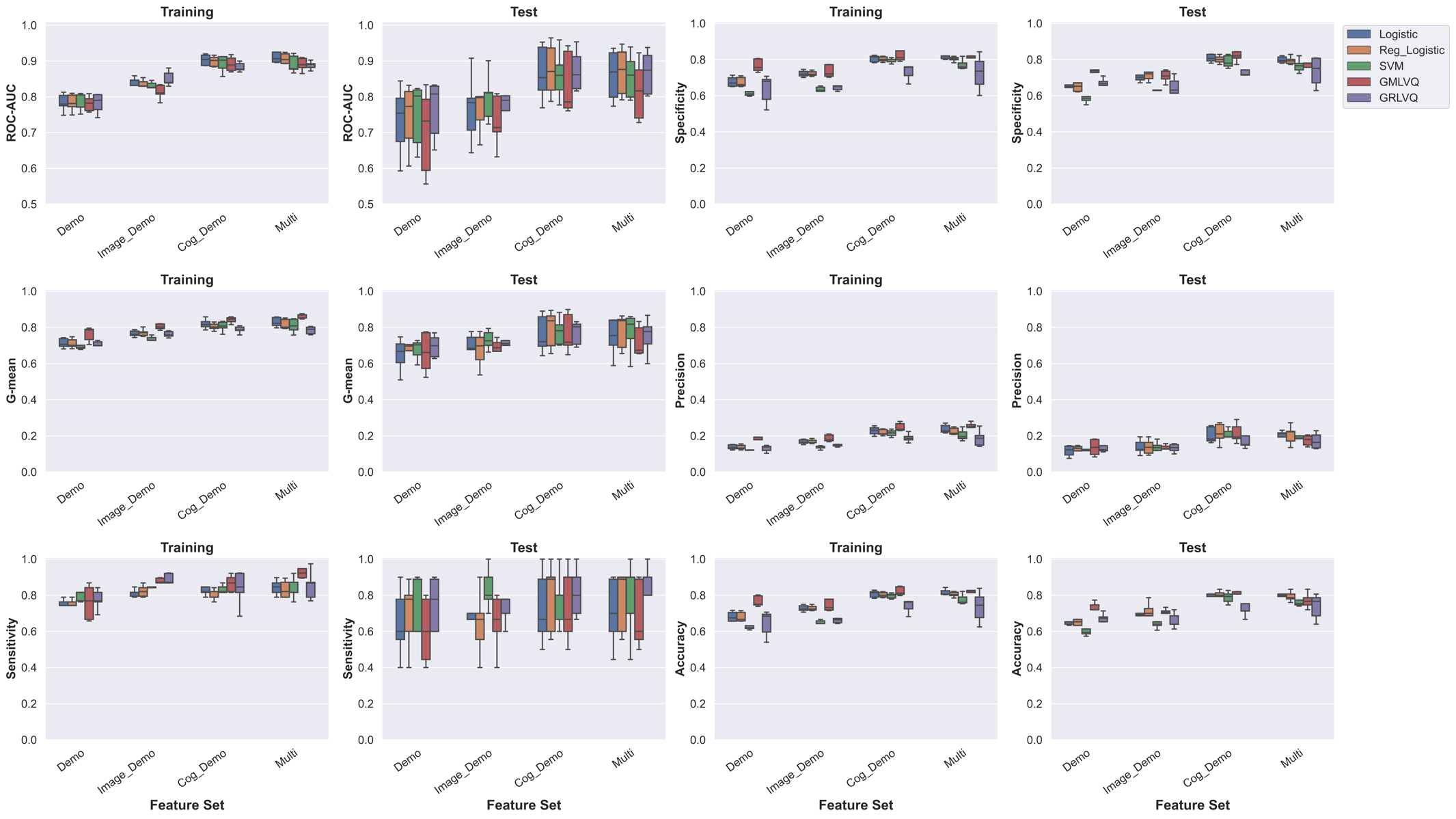


**Classification models**: Logistic = Logistic regression; Reg_Logistic = Regularised logistic regression with elastic net penalty; SVM = Support vector machine; GMLVQ = Generalised matrix learning vector quantisation; GRLVQ = Generalised relevance learning vector quantisation.

**Feature sets**: Demo = Demographic feature set (N=7); Image_Demo = Imaging and demographic feature set (N=12); Cog_Demo = Cognitive and demographic feature set (N=10); Multi = Multimodal feature set (N=15).

**Metrics**: ROC-AUC = Area under the receiver operating curve.

**Supplemental Tables**

**Table S1** – Transparent Reporting of a Multivariable Prediction Model for Individual Prognosis or Diagnosis (TRIPOD) Checklist^8^

| **Section/Topic** | **Item** |  | **Checklist Item** | **Page** |
| --- | --- | --- | --- | --- |
| **Title and abstract** | | | | |
| Title | 1 | D;V | Identify the study as developing and/or validating a multivariable prediction model, the target population, and the outcome to be predicted. | 0 |
| Abstract | 2 | D;V | Provide a summary of objectives, study design, setting, participants, sample size, predictors, outcome, statistical analysis, results, and conclusions. | 1,2 |
| **Introduction** | | | | |
| Background and objectives | 3a | D;V | Explain the medical context (including whether diagnostic or prognostic) and rationale for developing or validating the multivariable prediction model, including references to existing models. | 3 |
|  | 3b | D;V | Specify the objectives, including whether the study describes the development or validation of the model or both. | 4 |
| **Methods** | | | | |
| Source of data | 4a | D;V | Describe the study design or source of data (e.g., randomized trial, cohort, or registry data), separately for the development and validation data sets, if applicable. | 4,5 |
|  | 4b | D;V | Specify the key study dates, including start of accrual; end of accrual; and, if applicable, end of follow-up. | 22 |
| Participants | 5a | D;V | Specify key elements of the study setting (e.g., primary care, secondary care, general population) including number and location of centres. | 22 |
|  | 5b | D;V | Describe eligibility criteria for participants. | 22 |
|  | 5c | D;V | Give details of treatments received, if relevant. | N/A |
| Outcome | 6a | D;V | Clearly define the outcome that is predicted by the prediction model, including how and when assessed. | 6 |
|  | 6b | D;V | Report any actions to blind assessment of the outcome to be predicted. | N/A |
| Predictors | 7a | D;V | Clearly define all predictors used in developing or validating the multivariable prediction model, including how and when they were measured. | 5,6, Supp:1,14 |
|  | 7b | D;V | Report any actions to blind assessment of predictors for the outcome and other predictors. | N/A |
| Sample size | 8 | D;V | Explain how the study size was arrived at. | 6 |
| Missing data | 9 | D;V | Describe how missing data were handled (e.g., complete-case analysis, single imputation, multiple imputation) with details of any imputation method. | 6 |
| Statistical analysis methods | 10a | D | Describe how predictors were handled in the analyses. | 6, Supp:14 |
|  | 10b | D | Specify type of model, all model-building procedures (including any predictor selection), and method for internal validation. | 7,8,9,10 |
|  | 10c | V | For validation, describe how the predictions were calculated. | 9; Supp: 7,8 |
|  | 10d | D;V | Specify all measures used to assess model performance and, if relevant, to compare multiple models. | 8,9 |
|  | 10e | V | Describe any model updating (e.g., recalibration) arising from the validation, if done. | N/A |
| Risk groups | 11 | D;V | Provide details on how risk groups were created, if done. | N/A |
| Development vs. validation | 12 | V | For validation, identify any differences from the development data in setting, eligibility criteria, outcome, and predictors. | 9 |
| **Results** | | | | |
| Participants | 13a | D;V | Describe the flow of participants through the study, including the number of participants with and without the outcome and, if applicable, a summary of the follow-up time. A diagram may be helpful. | 11, 23 |
|  | 13b | D;V | Describe the characteristics of the participants (basic demographics, clinical features, available predictors), including the number of participants with missing data for predictors and outcome. | 23, 24, Supp: 17 |
|  | 13c | V | For validation, show a comparison with the development data of the distribution of important variables (demographics, predictors and outcome). | N/A  (cross validation) |
| Model development | 14a | D | Specify the number of participants and outcome events in each analysis. | 23 |
|  | 14b | D | If done, report the unadjusted association between each candidate predictor and outcome. | N/A |
| Model specification | 15a | D | Present the full prediction model to allow predictions for individuals (i.e., all regression coefficients, and model intercept or baseline survival at a given time point). | N/A |
|  | 15b | D | Explain how to the use the prediction model. | N/A |
| Model performance | 16 | D;V | Report performance measures (with CIs) for the prediction model. | 25, 26, Supp: 20,21,22 |
| Model-updating | 17 | V | If done, report the results from any model updating (i.e., model specification, model performance). | N/A |
| **Discussion** | | | | |
| Limitations | 18 | D;V | Discuss any limitations of the study (such as nonrepresentative sample, few events per predictor, missing data). | 15, 16 |
| Interpretation | 19a | V | For validation, discuss the results with reference to performance in the development data, and any other validation data. | 12, Supp: 9,10 |
|  | 19b | D;V | Give an overall interpretation of the results, considering objectives, limitations, results from similar studies, and other relevant evidence. | 14, 15 |
| Implications | 20 | D;V | Discuss the potential clinical use of the model and implications for future research. | 15, 16 |
| **Other information** | | | | |
| Supplementary information | 21 | D;V | Provide information about the availability of supplementary resources, such as study protocol, Web calculator, and data sets. | Supplementary Materials |
| Funding | 22 | D;V | Give the source of funding and the role of the funders for the present study. | 17, COI forms |

**Table S2** – Complete list of selected input features for dementia prediction models

| **Modality** | **Feature** | **Abbreviation** | **Explanation** |
| --- | --- | --- | --- |
| **Demographic** | Age | Age | Age in years. |
|  | Sex | Sex | Binary variable. Male or female. |
|  | Years of education | Education | - |
|  | Hypertension | HTN | Binary variable. On medication was take as "yes". |
|  | Smoking | Smoking | Binary variable. "Ever"="yes"; "never"="no". |
|  | Diabetes mellitus | Diabetes | Binary variable. On medication was take as "yes". |
|  | Hypercholesterolemia | HC | Binary variable. On medication was take as "yes". |
| **Imaging** | Total brain volume | TBV | Sum of the volume of white matter and grey matter as segmented by dedicated software. Measured in ml. |
|  | White matter lesion load | WMLL | Calculated as WMH volume ÷ total brain volume×100%. In practice, we used ${log}_{e}(WMLL+0.005)$ to adjust for severe right skewness. |
|  | Lacune count | Lacunes | Number of lacunes or lacunar infarcts, which was manually counted by trained independent raters according to published neuroimaging standards.^9^ |
|  | Presence of cerebral microbleeds | CMB | Binary variable. Cerebral microbleeds were identified manually by trained raters. |
|  | Peak width of skeletonised mean diffusivity | PSMD | A fully automatic marker derived from DTI measuring white matter ultrastructural damage. In practice, we used ${log}_{e}(PSMD)$ to avoid arithmetic underflow. |
| **Cognitive** | Global cognition | Global | A summary score over multiple cognitive domains. Calculated as the average of the z-scores from all neuropsychological tests used in each cohort. |
|  | Executive function | EF | A cognitive index score for the executive function domain. Calculated as the average of the z-scores from tests measuring this domain. |
|  | Processing speed | PS | A cognitive index score for the processing speed domain. Calculated as the average of the z-scores from tests measuring this domain. |
|  |  |  |  |

WMH = White matter hyperintensity; DTI = Diffusion tensor imaging.

**Table S3** – Neuropsychological assessment batteries used in each cohort

| **Cohort** | **Cognitive Domain** | **Name of Neuropsychological Test and Brief Details** |
| --- | --- | --- |
| **RUN DMC**^10^ | Executive Function | - Verbal Fluency Test (animals and profession naming) - Stroop Colour Word Test (short form) - Verbal Series Attention Test (include forward and reverse generation of arithmetic series, days of the week, and months of the year; number-letter sequencing; and auditory vigilance for a spoken target letter. |
|  | Processing Speed | - Paper-Pencil Memory Scanning Task - Symbol Digit Substitution Task (involving match symbols to numbers according to a key) |
|  | Memory | - Rey Auditory Verbal Learning Test (3-trial version) |
|  |  |  |
| **SCANS**^1^ | Executive Function | - Trail-making Test-B (time to complete part B) - Modified Wisconsin Card Sorting Test, average of categories achieved and preservative errors - Verbal fluency, total number of correct words generated |
|  | Processing Speed | - BIRT Memory and Information Processing Battery - Digit symbol Substitution Test, total score - Grooved Pegboard Test, best of left or right hand |
|  | Memory | - Digit Span - Logical Memory from Wechsler Memory Scale-III, average of immediate and delayed recall - Visual Reproduction from Wechsler Memory Scale-III, average of immediate and delayed recall |
|  |  |  |
| **HARMONISATION**^11^ | Executive Function | - Frontal Assessment Battery |
|  | Visuomotor Speed (used for Processing Speed) | - Symbol Digit Modality Test - Digit Cancellation Task - Maze Task |
|  | Visuo-construction | - Weschler Memory Scale—Revised (Visual Reproduction Copy task) - Clock Drawing Task - Weschler Adult Intelligence Scale—Revised subtest of Block Design |
|  | Verbal Memory | - Word List Recall Task - Story Recall Task |
|  | Visual Memory | - Picture Recall Task - Weschler Memory Scale—Revised (Immediate and Delayed Recall and Delayed Recognition Task) |
|  | Attention | - Digit Span Task - Visual Memory Span Task - Auditory Detection Task |
|  | Language | - Boston Naming Test - Verbal Fluency Test |

**Table S4** – The number of subjects missing each variable in each cohort and the pooled dataset

| **Cohort** | **RUN DMC** | **SCANS** | **HARMONISATION** | **POOLED** |
| --- | --- | --- | --- | --- |
| Baseline cohort size | 503 | 121 | 265 | 889 |
| No. complete cases | 439 (87.3%) | 110 (90.9%) | 240 (90.6%) | 789 (88.9%) |
| *Variable* | *N missing* | *N missing* | *N missing* | *N missing* |
| Age | 0 | 0 | 23 | 23 |
| Sex | 0 | 0 | 23 | 23 |
| Years of education | 0 | 8 | 23 | 31 |
| Hypertension | 0 | 0 | 23 | 23 |
| Smoking | 0 | 0 | 23 | 23 |
| Diabetes mellitus | 0 | 0 | 23 | 23 |
| Hypercholesterolemia | 0 | 0 | 23 | 23 |
| Total brain volume | 0 | 1 | 23 | 24 |
| White matter lesion load | 0 | 1 | 23 | 24 |
| Lacune count | 0 | 0 | 23 | 23 |
| Presence of cerebral microbleeds | 4 | 0 | 24 | 28 |
| PSMD | 59 | 1 | 23 | 83 |
| Global cognition | 0 | 0 | 1 | 1 |
| Executive function | 0 | 1 | 0 | 1 |
| Processing speed | 1 | 2 | 1 | 4 |
| Dementia Outcome | 2 | 0 | 0 | 2 |

PSMD = Peak width of skeletonised mean diffusivity

**Table S5** – Implementation details of the survival and classification models

| **Model Type** | **Model** | **Hyperparameter Search Range** | **Package** | **Function** |
| --- | --- | --- | --- | --- |
| Survival Model | CoxPH | alpha=0.0 | scikit-survival 0.17.2^12^ | linear_model.  CoxPHSurvivalAnalysis |
|  | Reg_Cox | alphas = [0.001 🡪 1]  l1_ratio = [0.2, 0.4, 0.6, 0.8, 1.0] | scikit-survival 0.17.2^12^ | linear_model.  CoxnetSurvivalAnalysis |
|  | RSF | n_estimators = [50, 100, 150]  max_depth = [5, 7, 9]  max_features = ‘sqrt’ | scikit-survival 0.17.2^12^ | ensemble.  RandomSurvivalForest |
|  | GBT | n_estimators = [150, 175, 200, 225, 250]  max_depth = 1  max_features = ‘sqrt’  subsample = 0.2 | scikit-survival 0.17.2^12^ | ensemble.GradientBoostingSurvivalAnalysis |
| Classification Model | Logistic | – | scikit-learn 1.0.2^13^ | linear_model.LogisticRegression |
|  | Reg_Logistic | penalty = ‘elasticnet’  C = [0.1, 0.5, 1, 2]  l1_ratio = [0.1, 0.3, 0.5, 0.7, 0.9]  solver = ‘saga’ | scikit-learn 1.0.2^13^ | linear_model.LogisticRegression |
|  | SVM | C = [0.2, 0.4, 0.6, 0.8, 1.0]  kernel = [‘linear’, ‘rbf’]  gamma = [10^-7^, 10^-6^, 10^-5^, 10^-4^, 10^-3^, 10^-2^, 10^-1^, 1] | scikit-learn 1.0.2^13^ | svm.SVC |
|  | GMLVQ | solver_type = ‘bfgs’  activation_type = ‘soft+’  activation_param_beta = 1  number of prototypes for class 0 = [1, 3, 5, 7]  number of prototypes for class 1 = [1, 3, 5, 7] | sklvq 0.1.2^14^ | GMLVQ |
|  | GRLVQ | number of prototypes for class 0 = [1, 3, 5, 7]  number of prototypes for class 1 = [1, 3, 5, 7] | sklearn_lvq 1.1.1^*^ | GrlvqModel |

^*^Package at https://github.com/MrNuggelz/sklearn-lvq.

N.B. Default settings were used for any hyperparameters not mentioned above, including cut-off thresholds.

**Survival models**: CoxPH = Cox proportional hazards model; Reg_Cox = Regularised CoxPH model with elastic net penalty; RSF = Random survival forests; GBT = Gradient boosted survival trees.

**Classification models**: Logistic = Logistic regression; Reg_Logistic = Regularised logistic regression with elastic net penalty; SVM = Support vector machine; GMLVQ = Generalised matrix learning vector quantisation; GRLVQ = Generalised relevance learning vector quantisation.

**Table S6** – Full test results from the 5-fold nested cross-validation experiments in survival analysis.

| **Metric** | **Model** | **Feature Set** | | | | |
| --- | --- | --- | --- | --- | --- | --- |
|  |  | **Demo** | **Image_Demo** | **Cog_Demo** | **Multi** |  |
| C-Index | CoxPH | 0.756 (0.051) | **0.801 (0.057)** | 0.859 (0.060) | 0.858 (0.055) |  |
|  | Reg_Cox | **0.757 (0.051)** | 0.799 (0.056) | **0.861 (0.058)** | **0.863 (0.053)** |  |
|  | RSF | 0.746 (0.044) | 0.777 (0.064) | 0.845 (0.053) | 0.848 (0.046) |  |
|  | GBT | 0.756 (0.051) | 0.796 (0.051) | 0.840 (0.058) | 0.844 (0.046) |  |
|  |  |  |  |  |  |  |
| IBS | CoxPH | **0.075 (0.010)** | **0.071 (0.008)** | **0.056 (0.012)** | 0.056 (0.012) |  |
|  | Reg_Cox | **0.075 (0.010)** | 0.072 (0.007) | **0.056 (0.012)** | **0.056 (0.011)** |  |
|  | RSF | 0.078 (0.006) | 0.075 (0.006) | 0.061 (0.008) | 0.060 (0.007) |  |
|  | GBT | 0.078 (0.008) | 0.073 (0.005) | 0.062 (0.009) | 0.061 (0.007) |  |

N.B. Results are presented in *mean (standard deviation).*

The best mean result among 4 models in each metric for each feature set is labelled in **bold** – where means are equal, the result with a smaller standard deviation was chosen as the better one.

**Survival models**: CoxPH = Cox proportional hazards model; Reg_Cox = Regularised CoxPH model with elastic net penalty; RSF = Random survival forests; GBT = Gradient boosted survival trees.

**Feature sets**: Demo = Demographic feature set (N=7); Image_Demo = Imaging and demographic feature set (N=12); Cog_Demo = Cognitive and demographic feature set (N=10); Multi = Multimodal feature set (N=15).

**Metrics**: C-index = Concordance index; IBS = Integrated brier score.

**Table S7** – Full test results from the 5-fold nested cross-validation experiments in classification analysis.

| **Metric** | **Model** | **Feature Set** | | | |
| --- | --- | --- | --- | --- | --- |
|  |  | **Demo** | **Image_Demo** | **Cog_Demo** | **Multi** |
| ROC-AUC | Logistic | 0.732 (0.089) | 0.768 (0.089) | 0.867 (0.070) | 0.860 (0.065) |
|  | Reg_Logistic | 0.742 (0.085) | 0.782 (0.080) | **0.876 (0.067)** | **0.870 (0.061)** |
|  | SVM | 0.749 (0.081) | **0.799 (0.062)** | 0.861 (0.062) | 0.858 (0.057) |
|  | GMLVQ | 0.702 (0.109) | 0.732 (0.066) | 0.837 (0.080) | 0.817 (0.075) |
|  | GRLVQ | **0.764 (0.075)** | 0.782 (0.079) | 0.873 (0.053) | 0.868 (0.055) |
|  |  |  |  |  |  |
| G-mean | Logistic | 0.648 (0.084) | 0.684 (0.083) | 0.762 (0.096) | 0.745 (0.094) |
|  | Reg_Logistic | 0.670 (0.082) | 0.676 (0.087) | **0.790 (0.095)** | **0.778 (0.087)** |
|  | SVM | 0.677 (0.050) | **0.731 (0.047)** | 0.778 (0.068) | 0.770 (0.103) |
|  | GMLVQ | 0.661 (0.102) | 0.672 (0.070) | 0.768 (0.099) | 0.724 (0.076) |
|  | GRLVQ | **0.695 (0.056)** | 0.706 (0.057) | 0.770 (0.059) | 0.751 (0.091) |
| Accuracy | Logistic | 0.663 (0.036) | 0.711 (0.038) | 0.803 (0.020) | **0.793 (0.017)** |
|  | Reg_Logistic | 0.665 (0.039) | **0.717 (0.038)** | 0.800 (0.024) | 0.793 (0.025) |
|  | SVM | 0.612 (0.043) | 0.656 (0.048) | 0.788 (0.028) | 0.768 (0.031) |
|  | GMLVQ | **0.725 (0.035)** | 0.704 (0.022) | **0.812 (0.026)** | 0.771 (0.038) |
|  | GRLVQ | 0.657 (0.049) | 0.660 (0.038) | 0.741 (0.065) | 0.737 (0.063) |
| Sensitivity | Logistic | 0.647 (0.175) | 0.667 (0.159) | 0.731 (0.185) | 0.707 (0.174) |
|  | Reg_Logistic | 0.693 (0.174) | 0.644 (0.165) | 0.789 (0.177) | 0.769 (0.157) |
|  | SVM | **0.776 (0.143)** | **0.836 (0.104)** | 0.773 (0.137) | 0.789 (0.198) |
|  | GMLVQ | 0.604 (0.165) | 0.649 (0.144) | 0.733 (0.187) | 0.689 (0.171) |
|  | GRLVQ | 0.753 (0.132) | 0.771 (0.132) | **0.813 (0.124)** | **0.789 (0.188)** |
|  |  |  |  |  |  |
| Specificity | Logistic | 0.664 (0.041) | 0.714 (0.042) | 0.808 (0.020) | **0.799 (0.016)** |
|  | Reg_Logistic | 0.664 (0.047) | **0.722 (0.041)** | 0.801 (0.020) | 0.795 (0.020) |
|  | SVM | 0.601 (0.052) | 0.644 (0.052) | 0.789 (0.031) | 0.766 (0.033) |
|  | GMLVQ | **0.734 (0.028)** | 0.708 (0.030) | **0.818 (0.026)** | 0.776 (0.048) |
|  | GRLVQ | 0.651 (0.057) | 0.652 (0.042) | 0.737 (0.072) | 0.733 (0.072) |
|  |  |  |  |  |  |
| Precision | Logistic | 0.116 (0.027) | 0.139 (0.036) | 0.205 (0.040) | 0.193 (0.041) |
|  | Reg_Logistic | 0.123 (0.024) | 0.138 (0.037) | 0.214 (0.051) | **0.205 (0.048)** |
|  | SVM | 0.117 (0.012) | **0.140 (0.023)** | 0.202 (0.035) | 0.189 (0.050) |
|  | GMLVQ | **0.136 (0.041)** | 0.130 (0.020) | **0.215 (0.048)** | 0.175 (0.026) |
|  | GRLVQ | 0.129 (0.014) | 0.132 (0.021) | 0.186 (0.060) | 0.172 (0.039) |

N.B. Results are presented in *mean (standard deviation).*

The best mean result among 5 models in each metric for each feature set was labelled in **bold** – where means are equal, the result with a smaller standard deviation was chosen as the better one.

**Classification models**: Logistic = Logistic regression; Reg_Logistic = Regularised logistic regression with elastic net penalty; SVM = Support vector machine; GMLVQ = Generalised matrix learning vector quantisation; GRLVQ = Generalised relevance learning vector quantisation.

**Feature sets:** Demo = Demographic feature set (N=7); Image_Demo = Imaging and demographic feature set (N=12); Cog_Demo = Cognitive and demographic feature set (N=10); Multi = Multimodal feature set (N=15).

**Metrics**: ROC-AUC = Area under the receiver operating curve.

**Table S8** – Full results for obtaining the feature importance rankings by survival models.

| **Ranking** | **CoxPH** | | **Reg_Cox** | | **RSF** | | **GBT** | |
| --- | --- | --- | --- | --- | --- | --- | --- | --- |
|  | **Feature** | **β coefficient** | **Feature** | **β coefficient** | **Feature** | **Δ C-index** | **Feature** | **Δ C-index** |
| 1 | Global Cog | -1.964 | Global Cog | -1.593 | Global Cog | -0.018 | Global Cog | -0.021 |
| 2 | Age | 0.658 | Age | 0.558 | Age | -0.014 | Age | -0.010 |
| 3 | PS | 0.506 | PS | 0.267 | HC | -0.007 | WMLL^*^ | -0.002 |
| 4 | Education | 0.331 | Education | 0.265 | Sex | -0.005 | HC^*^ | -0.002 |
| 5 | EF | 0.261 | TBV | -0.200 | CMB^*^ | -0.004 | TBV | 0.000 |
| 6 | TBV | -0.241 | PSMD | 0.163 | HTN^*^ | -0.004 | Education^*^ | 0.001 |
| 7 | PSMD | 0.208 | EF | 0.124 | TBV^†^ | -0.003 | Diabetes^*^ | 0.001 |
| 8 | Lacunes | 0.189 | Lacunes | 0.099 | PSMD^†^ | -0.003 | EF | 0.002 |
| 9 | CMB | -0.179 | WMLL^*^ | 0.000 | EF^†^ | -0.003 | Lacunes^*^ | 0.003 |
| 10 | HTN | -0.162 | CMB^*^ | 0.000 | Diabetes^*^ | -0.002 | PSMD^*^ | 0.003 |
| 11 | Diabetes | 0.123 | Sex^*^ | 0.000 | Smoking^*^ | -0.002 | CMB^†^ | 0.004 |
| 12 | HC | -0.106 | HTN^*^ | 0.000 | WMLL | -0.001 | PS^†^ | 0.004 |
| 13 | WMLL | -0.071 | HC^*^ | 0.000 | Lacunes | 0.000 | Sex^†^ | 0.004 |
| 14 | Smoking | -0.068 | Diabetes^*^ | 0.000 | PS | 0.001 | HTN^†^ | 0.004 |
| 15 | Sex | 0.047 | Smoking^*^ | 0.000 | Education | 0.003 | Smoking | 0.006 |

^*^ or ^†^ denotes tied ranking. Two symbols were used to distinguish adjacent tied rankings.

**Survival models**: CoxPH = Cox proportional hazards model; Reg_Cox = Regularised CoxPH model with elastic net penalty; RSF = Random survival forests; GBT = Gradient boosted survival trees.

**Features:** Global cog = Global cognition; PS = Processing speed; EF = Executive function; TBV = Total brain volume; PSMD = Peak width of skeletonised mean diffusivity; CMB = Cerebral microbleed; HTN = Hypertension; HC = Hypercholesterolemia; WMLL = White matter lesion load.

Values shown for standard/regularised Cox models are the β coefficient associated with each feature in the final models trained on the entire pooled dataset. Values shown for RSF and GBT are the average change in test c-index from nested cross validation experiments when each feature was excluded the complete feature set.

**Table S9** – Full results for obtaining the feature importance rankings by classification models.

| **Ranking** | **Logistic** | | **Reg_Logistic** | | **SVM** | | **GMLVQ** | | **GRLVQ** | |
| --- | --- | --- | --- | --- | --- | --- | --- | --- | --- | --- |
|  | **Feature** | **β coefficient** | **Feature** | **β coefficient** | **Feature** | **Δ ROC-AUC** | **Feature** | **Weight** | **Feature** | **Weight** |
| 1 | Global Cog | -2.757 | Global Cog | -1.862 | Global Cog | -0.018 | Global Cog | 0.534 | Global Cog | 0.617 |
| 2 | HC | -0.812 | HC | -0.689 | TBV | -0.007 | CMB | 0.109 | Age | 0.170 |
| 3 | Age | 0.619 | Age | 0.529 | PSMD | -0.002 | Age | 0.080 | EF | 0.097 |
| 4 | PS | 0.592 | PSMD | 0.378 | WMLL^*^ | -0.001 | Sex | 0.058 | TBV | 0.089 |
| 5 | EF | 0.587 | Education | 0.363 | CMB^*^ | -0.001 | PS | 0.051 | CMB | 0.019 |
| 6 | Education | 0.509 | TBV | -0.209 | PS^*^ | -0.001 | PSMD | 0.049 | Sex | 0.005 |
| 7 | HTN | -0.472 | EF | 0.206 | Age^*^ | -0.001 | HTN | 0.041 | Smoking | 0.003 |
| 8 | PSMD | 0.448 | PS | 0.184 | HTN^†^ | 0.000 | HC | 0.031 | Diabetes | 0.001 |
| 9 | Sex | 0.396 | Lacunes | -0.098 | HC^†^ | 0.000 | Smoking | 0.016 | WMLL^*^ | 0.000 |
| 10 | Smoking | 0.332 | CMB | -0.094 | Diabetes | 0.001 | EF | 0.008 | Lacunes^*^ | 0.000 |
| 11 | CMB | -0.323 | HTN | -0.088 | Smoking | 0.002 | Diabetes | 0.007 | PSMD^*^ | 0.000 |
| 12 | TBV | -0.320 | Sex | 0.025 | Sex | 0.003 | Education | 0.006 | PS^*^ | 0.000 |
| 13 | Diabetes | 0.200 | Smoking^*^ | 0.000 | Lacunes | 0.004 | TBV | 0.005 | Education^*^ | 0.000 |
| 14 | WMLL | 0.137 | Diabetes^*^ | 0.000 | EF | 0.005 | Lacunes | 0.004 | HTN^*^ | 0.000 |
| 15 | Lacunes | -0.078 | WMLL^*^ | 0.000 | Education | 0.009 | WMLL | 0.000 | HC^*^ | 0.000 |

^*^ or ^†^ denotes tied ranking. Two symbols were used to distinguish adjacent tied rankings.

**Classification models**: Logistic = Logistic regression; Reg_Logistic = Regularised logistic regression with elastic net penalty; SVM = Support vector machine; GMLVQ = Generalised matrix learning vector quantisation; GRLVQ = Generalised relevance learning vector quantisation.

**Features:** Global cog = Global cognition; PS = Processing speed; EF = Executive function; TBV = Total brain volume; PSMD = Peak width of skeletonised mean diffusivity; CMB = Cerebral microbleed; HTN = Hypertension; HC = Hypercholesterolemia; WMLL = White matter lesion load.

Values shown for standard/regularised logistic regression are the β coefficient associated with each feature in the final models trained on the entire pooled dataset. Values for SVM are the average change in test ROC-AUC from nested cross validation experiments when each feature was excluded from the complete feature set. Values shown for the LVQ models are the diagonal entries of the relevance matrices of the final LVQ models trained on the entire pooled dataset.

**References**

1. Lawrence AJ, Brookes RL, Zeestraten EA, et al. Pattern and Rate of Cognitive Decline in Cerebral Small Vessel Disease: A Prospective Study. *PLoS One* 2015; 10: e0135523.

2. Cox DR. Regression Models and Life-Tables. *Journal of the Royal Statistical Society: Series B (Methodological)* 1972; 34: 187–202.

3. Ishwaran H, Kogalur UB, Blackstone EH, et al. Random survival forests. *Annals of Applied Statistics* 2008; 2: 841–860.

4. Friedman JH. Greedy function approximation: A gradient boosting machine. *Ann Stat* 2001; 29: 1189–1232.

5. Cortes C. Support-Vector Networks. 1995; 20: 273–297.

6. Schneider P, Biehl M, Hammer B. Adaptive Relevance Matrices in Learning Vector Quantization. *Neural Comput* 2009; 21: 3532–3561.

7. Cawley GC, Talbot NLC. On Over-fitting in Model Selection and Subsequent Selection Bias in Performance Evaluation. *Journal of Machine Learning Research* 2010; 11: 2079–2107.

8. Collins GS, Reitsma JB, Altman DG, et al. Transparent reporting of a multivariable prediction model for individual prognosis or diagnosis (TRIPOD): The TRIPOD Statement. *BMC Med* 2015; 13: 1–10.

9. Wardlaw JM, Smith EE, Biessels GJ, et al. Neuroimaging standards for research into small vessel disease and its contribution to ageing and neurodegeneration. *Lancet Neurol* 2013; 12: 822–838.

10. van Uden IWM, van der Holst HM, Schaapsmeerders P, et al. Baseline white matter microstructural integrity is not related to cognitive decline after 5 years: The RUN DMC study. *BBA Clin* 2015; 4: 108–114.

11. Hilal S, Chai YL, Ikram MK, et al. Markers of cardiac dysfunction in cognitive impairment and dementia. *Medicine* 2015; 94: e297.

12. Pölsterl S. scikit-survival: A Library for Time-to-Event Analysis Built on Top of scikit-learn. *Journal of Machine Learning Research* 2020; 21: 1–6.

13. Pedregosa F, Varoquaux G, Gramfort A, et al. Scikit-learn: Machine Learning in Python. *Journal of Machine Learning Research* 2011; 12: 2825–2830.

14. van Veen R, Biehl M, de Vries G-J. sklvq: Scikit Learning Vector Quantization. *Journal of Machine Learning Research* 2021; 22: 1–6.
